# Equity in Genomic Medicine

**DOI:** 10.1146/annurev-genom-112921-022635

**Published:** 2022-04-01

**Authors:** Chanita Hughes Halbert

**Affiliations:** 1 Department of Population and Public Health Sciences, University of Southern California, Los Angeles, California, USA; 2 Norris Comprehensive Cancer Center, University of Southern California, Los Angeles, California, USA

**Keywords:** equity, genomic medicine, minority health, translational research

## Abstract

Since the completion of the Human Genome Project, considerable progress has been made in translating knowledge about the genetic basis of disease risk and treatment response into clinical services and public health interventions that have greater precision. It is anticipated that more precision approaches to early detection, prevention, and treatment will be developed and will enhance equity in healthcare and outcomes among disparity populations. Reduced access to genomic medicine research, clinical services, and public health interventions has the potential to exacerbate disparities in genomic medicine. The purpose of this article is to describe these challenges to equity in genomic medicine and identify opportunities and future directions for addressing these issues. Efforts are needed to enhance access to genomic medicine research, clinical services, and public health interventions, and additional research that examines the clinical utility of precision medicine among disparity populations should be prioritized to ensure equity in genomic medicine.

## INTRODUCTION

The completion of the Human Genome Project is one of the most significant scientific accomplishments of the twenty-first century (13, 22). As a result of this research, novel genomic approaches for disease prevention, treatment, management, and health promotion have been developed and are being implemented in clinical care. Genetic counseling and testing for *BRCA1* and *BRCA2* (*BRCA1*/*2*) mutations is perhaps one of the best examples of genetically based healthcare and translational research in genomic medicine. Findings from basic science and epidemiological research have established the analytical validity of testing for *BRCA1*/*2* mutations (12), and psychosocial and behavioral studies have examined the clinical validity and utility of methods and strategies for identifying individuals at risk for hereditary disease and providing genetic test results (49, 73, 74). Genetic counseling and testing is now standard of care for individuals who have a personal or family history of breast and/or ovarian cancer that is suggestive of hereditary disease. Relatedly, genomic sequencing of tumors is now being integrated into cancer care to identify the most effective treatment strategies. For instance, before the availability of genomic sequencing for cancer tumors, it was difficult to predict which patients were likely to respond to immunotherapy. With next-generation sequencing, however, it is now possible to screen tumors for prognostic and predictive biomarkers to inform treatment decisions for some types of cancers. For non-small-cell lung cancer, for instance, *EGFR* mutation analysis and PD-L1 testing are recommended for patients who have advanced-stage disease (60). Even with these clinical advances and the ability to predict treatment responses based on the genomic profile of the tumor, African Americans continue to experience excess rates of morbidity and mortality from lung cancer (2).

Precision medicine strategies, especially those that are based on immunotherapy, target many patient-level factors, and this approach is expected to reduce racial disparities in health outcomes. Previous studies have examined differences in the prevalence of *EGFR* mutations between African Americans and whites to provide insight into how genetic factors may contribute to racial disparities in lung cancer outcomes. Early findings from this research demonstrated that the frequency of somatic *EGFR* mutations was similar between African American and white non-small-cell lung cancer patients (16), but results from a recent meta-analysis of targetable mutations in diverse lung cancer patients demonstrated that the prevalence of *EGFR* mutations was 6% in Black patients, 12% in white patients, 35% in Hispanic patients, and 46% in Asian patients (15). However, patients may be offered *EGFR* testing based on the likelihood that they carry a mutation using clinical factors (e.g., smoking history and race), and only five of the studies included in the meta-analysis conducted by Costa et al. (15) included data on the prevalence of *EGFR* mutations in African American patients. Furthermore, Lynch et al. (51) found that *EGFR* testing is underutilized among African American patients and that the decision to offer this testing may be based on social factors that include race, gender, and health insurance status. Using data from the National Cancer Database, Verma et al. (82) examined utilization of immunotherapy among newly diagnosed metastatic lung cancer patients and found that, in addition to geographic factors (e.g., travel distance to treatment facilities), treatment factors, and area-level education, utilization of immunotherapy-type compounds was lower among African American patients regardless of their insurance coverage.

Precision medicine strategies will be effective at addressing racial disparities in healthcare and outcomes only if studies that use genomic sequencing have adequate representation of racial minorities and if the clinical utility of precision medicine strategies, which encompasses the psychosocial and behavioral impact of genomic information, is evaluated in disparity populations. Moreover, for historically underserved and under-resourced communities and populations, challenges related to limited infrastructure for genomic sequencing and institutional access to emerging technologies may be barriers to the integration of genomic services into practice. It is important to identify and address barriers to the implementation of genomic services in healthcare in clinical settings that are representative of the types of practices where minorities and medically underserved patients are likely to receive medical care. Without attention to these issues, precision medicine that is based on genomic information has the potential to exacerbate racial disparities in healthcare and outcomes. As investments continue to be made to improve the precision of healthcare through initiatives that focus on integrating genomic information with lifestyle factors and environmental determinants (87), it is important to understand challenges to equity in genomic medicine and identify opportunities to address these issues.

The purpose of this article is to describe challenges and identify opportunities for achieving equity in genomic medicine. Although the US population includes many disparity populations, African Americans were among the first populations to be identified as a priority for genomic medicine and research, and early studies focused on addressing barriers to African American participation in genomic research and developing interventions to address the priorities and concerns of this population. Accordingly, this article describes primary challenges to equity in genomic medicine and identifies priorities and opportunities for ensuring equity in genomics-based healthcare using examples from previous research with African Americans. These examples are used to illustrate key findings from genomic medicine research to identify issues that should be prioritized to ensure equity in genomic medicine.

## REPRESENTATION OF DISPARITY POPULATIONS IN GENOMIC MEDICINE RESEARCH

Ensuring the adequate representation of racial and ethnic minorities in cancer research is a national and local priority; several criteria may be used to determine whether the inclusion of ethnic and racial minorities in medical research is adequate (14). These criteria include whether study samples reflect the proportion of racial and ethnic minorities in the general population or catchment area, whether the sample is similar to the distribution of disease risk in the population, and whether a sufficient number of racial and ethnic minorities have been enrolled to allow subgroup analyses of treatment effects to be performed. These criteria have different implications for the number of racial and ethnic minorities who are ultimately enrolled and can raise several important ethical principles. For instance, the National Cancer Institute now requires centers to demonstrate how their research addresses the cancer burden in their catchment area (beneficence), and the representation of women, children, and minorities (justice) is reviewed to determine National Cancer Institute center designation.

Consistent with this, previous efforts have focused on enrolling disparity populations into resources that were developed to support genomic research. For example, the Cancer Genetics Network was established by the National Cancer Institute to conduct collaborative studies on genetic risk factors for cancer in samples that were representative of the US population (10). Despite recruitment at geographically dispersed centers throughout the United States, Cancer Genetics Network centers experienced significant challenges recruiting disparity populations into the registry (42), and less than 10% of individuals enrolled in the network were from ethnic and racial minority groups (3), making the network registry unrepresentative of the US population.

Lower recruitment yields are at the heart of the limited participation of disparity populations in genomic research. Data from one Cancer Genetics Network center demonstrated that African Americans (15%) were significantly less likely than whites (36%) to enroll in the registry (58). A study that examined the representation of disparity populations in the Cancer Genome Atlas in July 2015 found that 12% (of 5,729 samples) were from African Americans, 3% were from Hispanics, and 77% were from whites, but the number of patients from disparity populations at this point was not sufficient to detect common genomic alterations in racial and ethnic minorities (78). Similarly, Popejoy & Fullerton (65) found that racial and ethnic minorities continued to be underrepresented in the GWAS Catalog from 2009 to 2016: Less than 4% of samples included in the catalog in 2016 were from individuals of African, Latin American, or Hispanic ancestry. In more recent research, Halbert et al. (27) demonstrated that African American prostate cancer patients were less likely than white prostate cancer patients to participate in a social determinants survey study that was completed as part of a precision medicine center in minority men’s health. Regardless of racial background, greater social deprivation was associated with a reduced likelihood of study enrollment (27).

Adequate representation of minorities in genomic research is needed to understand how genetic variants are relevant to disease risk and outcomes in diverse populations. Just as important, it is necessary for racial and ethnic minorities to be included in genomic medicine studies to understand the ethical, legal, and social implications of this technology within and among populations. For this reason, it is also necessary to understand recruitment and retention outcomes based on attitudes about genomic medicine and barriers and facilitators for participating in genetic research. McDonald et al. (56, 57) demonstrated that African Americans in a national sample (*n* = 1,033) have both positive and negative attitudes about genetic research that are important to intentions to participate in cancer genetics research, contribute to a biobank, or enroll in a study that focuses on precision medicine. For instance, the top three barriers to participation in cancer genetics research among participants in this study included concerns about who would be able to access one’s personal information, difficulty in getting to where the study was being conducted, and lack of access to findings of the study. The top three facilitators of participation in cancer genetics research included the availability of free medication or healthcare for participants, the study addressing a health condition that was personally relevant, and study participation lasting a short period of time. With respect to beliefs about genetically targeted medical care, 49% of African American patients in a hospital-based sample (*n* = 206) believed that this type of care would limit access to medical treatment, 46% believed that people would not trust it, and 20% believed that people like them would not benefit from it (32). However, only 31% of African Americans in a national sample (*n* = 510) reported that they were likely to participate in a study that involved donating a biospecimen that could be used for future research, completing a questionnaire, and not receiving information about one’s personal test result (33).

## ACCESS TO GENOMIC MEDICINE IN DISPARITY POPULATIONS

As we move into a new era of genomic medicine in which it is possible for individuals to obtain genetic risk information for multiple genes and chronic conditions simultaneously, it is critical to develop protocols and procedures for genomic medicine that are accessible to those at greatest risk, are consistent with the natural history and outcomes of the disease, and address the conceptualizations about disease and healthcare experiences of these populations, especially if genomic medicine is to play a role in reducing racial disparities in healthcare and outcomes. Existing methods for integrating genomic medicine into clinical care and public health practice have exacerbated racial disparities in access to care. For instance, most individuals who use *BRCA1*/*2* mutation testing are white and well educated and have high income levels (40, 48, 49, 75), since genetic counseling and testing are most often available in academic health centers that racial minorities are not able to access because of limited financial resources and lack of health insurance. Acceptance of genetic counseling for *BRCA1*/*2* mutations is lower among African Americans than among whites (4), possibly because of lower rates of provider referral to genetic testing among African American women (55). Similarly, in the Multiplex Initiative—a study supported by the National Human Genome Research Institute and the National Cancer Institute to examine how patients interpret and respond to test results from genetic panels and engage with the healthcare system to address this information (54)—enrollment rates were significantly lower among African Americans than among whites, interest in genetic testing was lower among African Americans than among whites, and only 30% of African Americans received multiplex testing, compared with 55% of whites (1).

Low rates of African American participation in genetic research and use of genomic medicine have been attributed to distrust of healthcare institutions and providers (4, 5), lack of knowledge about genetic testing and hereditary disease (38, 48), and psychosocial and cultural factors (37, 80). However, Halbert et al. (25, 30, 31) found that when efforts are made to move beyond traditional academic healthcare centers, and awareness about genetic testing in diverse settings is increased using appropriate strategies and resources, African Americans are likely to participate in genetic research and use genomic services.

Ensuring that individuals have appropriate content about genomic tests and testing outcomes has been a key consideration since the very beginning of translational efforts in genomic medicine (9). Genetic testing is evolving from being delivered in specialized high-risk counseling and testing programs to being delivered in primary care practice through multiplex testing and clinical decision support tools that are designed to identify and characterize risks for multiple diseases simultaneously (43, 62, 68). Family health history (FHH) is the basis for this type of genomic medicine (45, 81); national efforts encourage individuals to obtain their FHH and share it with their relatives and healthcare providers. Data from national and local studies show that individuals are not likely to have experience obtaining their FHH (35, 47) even though most believe that it is important to their health (6). African Americans have limited knowledge about basic concepts involved in genomic medicine (44) and applications of this knowledge to specific disease (38). In a sample of primary care patients from a community healthcare center, for instance, African Americans were less likely than whites to report that their FHH is important to their health overall and to be knowledgeable about their own FHH of disease (6). The results of the Family Healthware Impact Trial—a study conducted to determine the clinical utility and validity of FHH in a national sample of adults who did not have a personal history of disease—were positive and generated data on the clinical utility and psychological impact of FHH assessment and preventive risk messages (61, 70, 71); however, the study enrolled fewer than 150 African Americans, and these individuals made up only 4% of the study population even though more than 40% of African Americans have hypertension and African Americans (12.9%) are almost twice as likely as whites (7.6%) to be diagnosed with diabetes.

One strategy for increasing access to this type of genomic medicine among racial minorities is to integrate FHH tools into clinical practice; the Family Healthware Impact Trial demonstrated that this is feasible in primary care practices that are affiliated with academic health centers and have sufficient technology for electronic health records. However, many African Americans receive medical care in community health centers and federally qualified health centers that may not have the personnel or technological resources to support automated FHH assessment. Limited empirical data are available on the feasibility and impact of FHH collection and assessment in settings where medically underserved populations are likely to obtain health information and medical care services. Theisen et al. (79) distributed FHH forms in a faith-based organization as part of a community outreach program in an urban area but did not determine whether the forms were completed or assess the impact of FHH on health behaviors. Community education programs were also effective at addressing genetic literacy issues and prompting FHH collection among African Americans in a large metropolitan city (53). Nevertheless, there are limited examples of how to address the sociocultural needs and resources of racial minorities and other medically underserved groups within the context of genomic medicine, even though approaches that are sensitive to these issues are needed (8). Moreover, if populations with the greatest risk and disease burden are underrepresented in studies that were designed to evaluate the effects of the most basic form of genomic medicine, then the ethical, legal, and social consequences of current methods for implementing genomic tools and services in clinical care need to be examined.

## ETHICAL, LEGAL, AND SOCIAL CONSIDERATIONS FOR EQUITY IN GENOMIC MEDICINE

Several ethical, legal, and social issues have been considered as part of developing and delivering genetically informed care. Efforts have also been made to improve access to genomic testing offered as part of research protocols by oversampling some racial minority groups (1). But there are more fundamental ethical, legal, and social issues that should be addressed. First, knowledge about the genetic basis of disease is not based on racially diverse samples. Previous reports have shown that African Americans are underrepresented in genome-wide association studies (23, 65); therefore, in most cases, genetic variants associated with chronic diseases are first identified and validated in samples from white adults, and then replication studies are conducted in other populations. This approach is used to control for complex issues such as population stratification but is also necessary because racial minorities are not represented in existing data sets and cohorts at the same rates as whites (23, 58, 78). This hinders discovery and the translation of genomic discoveries into testing protocols and genomics-based strategies that are generalizable to disparity populations. For example, data on the prevalence of *BRCA1*/*2* mutations in racial minorities did not become available until 10 years after testing was first offered, and the significance of *BRCA1*/*2* mutations identified in these populations continues to be uncertain (59). Furthermore, it was not until 2009 that empirical data were available on the validity of a widely used risk estimation program for *BRCA1*/*2* mutations in racial minorities (41). Similar trends are now being observed for other conditions. Even though diabetes and hypertension are highly prevalent among African Americans, early research that was conducted to identify susceptibility genes for these conditions was based on samples from white adults with European ancestry. Studies are now being conducted to determine whether variants in these genes are present among African Americans at the same rate as in whites (11, 50).

Once the recruitment and retention of diverse populations have been addressed and these populations have sufficient access to emerging technology for genomic medicine, it is essential to evaluate the clinical utility of genomic information by examining the psychosocial and behavioral impact using patient-reported outcomes. However, very few studies have examined these outcomes in racially and ethnically diverse patient populations. One notable exception is research by Halbert et al. (25, 29) on genetic testing for *BRCA1*/*2* mutations in African American women. As part of the first randomized trial that compared culturally tailored and standard genetic counseling about *BRCA1*/*2* testing, this team examined uptake of genetic counseling and testing, measured the quality of informed decision-making about genetic testing (28), and evaluated the long-term impact of *BRCA1*/*2* test results (34). However, several important empirical questions remain as the technology for genetic testing advances and this technology is implemented in clinical care using new delivery models. For instance, during the first generation of research on genetic counseling and testing, pretest education and test results disclosure were completed by genetic counselors with training in cancer genetics and risk assessment (9). The second generation of studies examined alternative models of test results disclosure (e.g., telephone versus in person) in recognition of the limited number of genetic counselors nationally (46). Studies have also examined the effects of interventions that target genetic specific concerns (20). Research on clinical sequencing is also examining issues related to the return of results and psychological and behavioral issues associated with the disclosure of primary and secondary findings from whole-exome sequencing (21). This generation of research is incomplete, however, because racial and ethnic minorities are underrepresented in ongoing research. Importantly, empirical data are lacking on the clinical utility of whole-exome sequencing when it is integrated into a system of care for diseases where the quality of patient–physician communication may be suboptimal in disparity populations.

Regardless of the setting where genomic medicine is delivered, a critical outcome is that patients make informed decisions that are consistent with their personal and family values and preferences. This is perhaps one of the most critical ethical, legal, and social issues in genomic medicine. Halbert et al. (28) demonstrated that African American women are likely to make an informed decision about genetic testing for *BRCA1*/*2* mutations following both culturally tailored and standard genetic counseling. Other work has shown that there are no racial differences in specific concerns about long-term genetic testing, such as uncertainty about risk management and surveillance options (34). However, psychosocial, behavioral, and clinical outcomes may differ among cancer patients from medically underserved populations who have less exposure to and knowledge about information about genomic medicine (38, 44, 52).

As new technology for genomic medicine is introduced into clinical settings, it is critical to determine the short- and long-term psychosocial impact of this information in racially and ethnically diverse groups of patients. For instance, although many important clinical issues were considered as part of developing existing FHH tools, they may not address the sociocultural needs and resources of racial minority groups. Previous research has shown that African Americans do not base disease risk perceptions on objective clinical factors (e.g., personal or family history of disease) (39), and many cope with their FHH of disease by using cultural resources and beliefs (86) rather than clinical risk evaluation strategies. Furthermore, in community samples of African Americans, many did not believe that genetic factors are important to health or risk behaviors (26). Other research has also shown that tools for public health genomics may not be accessible to racial and ethnic minorities using existing delivery systems and methods. Wang et al. (83) found that many of the FHH tools do not meet the literacy needs of most adults, and these literacy issues may be compounded by the delivery systems for these tools; for example, the websites on which FHH tools are available may not be accessible by racial minorities. A previous national study found that African Americans have access to computer resources in their homes at significantly lower rates than whites; only 56% of African Americans in this study had used the internet to obtain health information (84).

Translational health informatics focuses on the translation of clinical and genomic data into individualized treatment and care through technology (76). It plays an essential role in precision medicine by integrating data across different sources and models; managing large, complex, heterogeneous data sets; supporting analysis and modeling; and providing access to data and results (18). Health informatics research has the potential to reduce racial disparities and improve healthcare delivery in diverse populations (19). However, the digital divide that exists in racial and ethnic minorities, along with the limited infrastructure for health information technology that exists in the settings where disparity populations typically receive medical care, may reduce access to precision medicine approaches that focus on integrating genomic data with clinical information, patient-reported data, and data on social determinants.

## OPPORTUNITIES AND FUTURE DIRECTIONS

It has now been more than a decade since the completion of the Human Genome Project (22); during this time, several genomic tools and services have been developed, evaluated, and implemented in clinical care. Many more personalized strategies for disease prevention and treatment are anticipated, and their value will ultimately be judged based on whether they can be translated into clinical care and public health practice and improve healthcare and outcomes, especially in medically underserved populations that are vulnerable to experiencing disparities in medical care and excess rates of morbidity and mortality from disease.

The examples provided in this article demonstrate that the racial disparities observed for healthcare and outcomes are perpetuated within the context of genomic medicine. The determinants of these disparities are complex, but one overarching factor is reduced access to care. Access to genomic medicine is not limited to whether someone has health insurance or other financial resources to pay for a particular genetic test. Rather, access is multifactorial and also based on whether an appropriate and valid tool is available and can be offered, whether individuals will accept or decline to use it, whether it is easy or difficult to use, whether individuals can get to the facilities where genomic tools and services are being offered, and the psychological and behavioral impact of interventions and services that are informed by genomic information (64). For this reason, ensuring equitable access to all phases of genomic medicine among racial minorities continues to be a high priority for future research and clinical practice.

Differences among racial groups in genetic test result acceptance have been a focus of previous investigations. Prior research on racial disparities and genomic research has addressed issues related to how studies were conducted and established recommendations for evaluating racial and ethnic group membership as part of genomic research (72, 77). Previous work has also concentrated on understanding the implications of genetics for the meaning of race and ethnicity (63, 67). At this point, however, the more critical issue is how to offer genomic tools and services to disparity populations in a way that maximizes their access to studies, clinical services, and public health interventions; enables them to provide informed consent; and lets them take advantage of benefits while minimizing exposure to the potential risks and harms of these strategies. Efforts should also focus on addressing access issues by translating valid and useful tools to these populations using methods and strategies that are sensitive to their sociocultural needs and resources. Accordingly, resources have been established to support genomic research in African American patient populations and to conduct genomic research among individuals at increased risk for chronic and acute conditions (7). For instance, the African American Hereditary Prostate Cancer Study Network was established in 2002 to identify genetic factors associated with prostate cancer in African American families (66, 69). More recently, the RESPOND (Research on Prostate Cancer in Men of African Ancestry: Defining the Roles of Genetics, Tumor Markers, and Social Stress; https://respondstudy.org) study was established to examine how the interactive effects of genetic factors, tumor characteristics, and stress exposure contribute to prostate cancer among African American men. Similarly, the Transdisciplinary Collaborative Center in Precision Medicine and Minority Men’s Health is examining the effects of stress responses on the initiation and progression of diseases among African American and white men (24, 36).

The inclusion of multilevel data on clinical, social, and environmental factors in translational studies and research resources is an important advance that is consistent with emerging conceptual models for minority health and health disparities (17). An expected outcome of obtaining and examining multilevel data related to minority health and health disparities is that these studies will generate novel findings on the complex ways in which individual-, interpersonal-, and community-level factors work independently and interact within the context of genomic medicine. However, it will be important to engage diverse stakeholders to translate discoveries from these multilevel data into policies, interventions, and services that are likely to be effective in terms of enhancing equity in genomic medicine. There are few examples of academic–community partnerships that specifically focus on genomic medicine, possibly because community stakeholders are most likely to be engaged in minority health and health disparities research that targets health promotion and disease prevention behaviors (85). As described in this article, ensuring adequate representation of disparity populations is important; community stakeholders have been engaged in some studies to address barriers to minority participation by providing input on recruitment and retention strategies (69). However, a greater level of community engagement is needed to identify and address disparity populations’ priorities and preferences for clinical services and public health interventions that are based on genomic information.

In addition to developing academic–community partnerships that have a specific focus on genomic medicine, it is critical to increase the diversity of genomic medicine researchers, clinicians, and public health providers. As part of increasing the diversity of the genomic medicine workforce, it is important to enhance the capacity of healthcare systems and practices that deliver medical care and public health interventions to disparity populations to provide genomic medicine services and interventions. It is also important to examine the short- and long-term effects of clinical and public health services and interventions among disparity populations in academic medical systems, community healthcare systems, and public health settings. Genetic counseling and testing for *BRCA1*/*2* mutations have been integrated into standard clinical practice as a result of the substantial evidence base that supported the clinical utility of counseling and testing without adverse psychological and behavioral effects. This evidence base has yet to be established for genomic medicine services and interventions in disparity populations and should be prioritized as part of future translational research to enhance equity in genomic medicine.

## References

[1] Alford SH, McBride CM, Reid RJ, Larson EB, Baxevanis AD, 2011. Participation in genetic testing research varies by social group. Public Health Genom. 14:85–93 10.1159/000294277 PMC3214933 20299772

[2] Am. Cancer Soc. 2019. Cancer facts and figures: 2019. Rep., Am. Cancer Soc, Atlanta

[3] Anton-Culver H, Ziogas A, Bowen D, Finkelstein D, Griffin C, 2003. The Cancer Genetics Network: recruitment results and pilot studies. Community Genet. 6:171–77 15237201 10.1159/000078165

[4] Armstrong K, Micco E, Carney A, Stopfer J, Putt M. 2005. Racial differences in the use of *BRCA1*/*2* testing among women with a family history of breast or ovarian cancer. JAMA 293:1729–36 15827311 10.1001/jama.293.14.1729

[5] Armstrong K, Weber B, Stopfer J, Calzone K, Putt M, 2003. Early use of clinical *BRCA1*/*2* testing: associations with race and breast cancer risk. Am. J. Med. Genet. A 117A:154–60 12567413 10.1002/ajmg.a.10928

[6] Ashida S, Goodman MS, Stafford J, Lachance C, Kaphingst KA. 2012. Perceived familiarity with and importance of family health history among a medically underserved population. J. Community Genet. 3:285–95 22569765 10.1007/s12687-012-0097-x PMC3461219

[7] Beebe-Dimmer JL, Albrecht TL, Baird TE, Ruterbusch JJ, Hastert T, 2019. The Detroit Research on Cancer Survivors (ROCS) pilot study: a focus on outcomes after cancer in a racially diverse patient population. Cancer Epidemiol. Biomark. Prev. 28:666–74 10.1158/1055-9965.EPI-18-0123 PMC6449184 30482875

[8] Berg AO, Baird MA, Botkin JR, Driscoll DA, Fishman PA, 2009. National Institutes of Health state-of-the-science conference statement: family history and improving health. Ann. Intern. Med. 151:872–77 19884615 10.7326/0003-4819-151-12-200912150-00165

[9] Biesecker BB, Boehnke M, Calzone K, Markel DS, Garber JE, 1993. Genetic counseling for families with inherited susceptibility to breast and ovarian cancer. JAMA 269:1970–74 8352830

[10] Bowen DJ, Vu T, Kasten-Sportes C. 2008. Increasing minority participant enrollment into a cancer family registry: the Cancer Genetics Network. Community Genet. 11:191–92 18417965 10.1159/000116877

[11] Bressler J, Kao WH, Pankow JS, Boerwinkle E. 2010. Risk of type 2 diabetes and obesity is differentially associated with variation in *FTO* in whites and African-Americans in the ARIC study. PLOS ONE 5:e10521 20502638 10.1371/journal.pone.0010521 PMC2873943

[12] Brody LC, Biesecker BB. 1998. Breast cancer susceptibility genes: BRCA1 and BRCA2. Medicine 77:208–26 9653432 10.1097/00005792-199805000-00006

[13] Collins FS, Green ED, Guttmacher AE, Guyer MS. 2003. A vision for the future of genomics research. Nature 422:835–47 12695777 10.1038/nature01626

[14] Corbie-Smith G, Miller WC, Ransohoff DF. 2004. Interpretations of ‘appropriate’ minority inclusion in clinical research. Am. J. Med. 116:249–52 14969653 10.1016/j.amjmed.2003.09.032

[15] Costa PA, Saul EE, Paul Y, Iyer S, da Silva LL, 2021. Prevalence of targetable mutations in Black patients with lung cancer: a systematic review and meta-analysis. JCO Oncol. Pract. 17:e629–36 33974815 10.1200/OP.20.00961

[16] Cote ML, Haddad R, Edwards DJ, Atikukke G, Gadgeel S, 2011. Frequency and type of epidermal growth factor receptor mutations in African Americans with non-small cell lung cancer. J. Thorac. Oncol. 6:627–30 21317742 10.1097/JTO.0b013e31820a0ec0 PMC3057407

[17] Dankwa-Mullan I, Rhee KB, Stoff DM, Pohlhaus JR, Sy FS, 2010. Moving toward paradigm-shifting research in health disparities through translational, transformational, and transdisciplinary approaches. Am. J. Public Health 100:S19–24 20147662 10.2105/AJPH.2009.189167 PMC2837422

[18] Frey LJ, Bernstam EV, Denny JC. 2016. Precision medicine informatics. J. Am. Med. Inform. Assoc. 23:668–70 27274018 10.1093/jamia/ocw053 PMC4926751

[19] Gibbons MC. 2005. A historical overview of health disparities and the potential of eHealth solutions. J. Med. Internet Res. 7:e50 16403714 10.2196/jmir.7.5.e50 PMC1550690

[20] Graves KD, Wenzel L, Schwartz MD, Luta G, Wileyto P, 2010. Randomized controlled trial of a psychosocial telephone counseling intervention in *BRCA1* and *BRCA2* mutation carriers. Cancer Epidemiol. Biomark. Prev. 19:648–54 10.1158/1055-9965.EPI-09-0548 PMC2849295 20200423

[21] Gray SW, Martins Y, Feuerman LZ, Bernhardt BA, Biesecker BB, 2014. Social and behavioral research in genomic sequencing: approaches from the Clinical Sequencing Exploratory Research Consortium Outcomes and Measures Working Group. Genet. Med. 16:727–35 24625446 10.1038/gim.2014.26 PMC4163120

[22] Green ED, Guyer MS. 2011. Charting a course for genomic medicine from base pairs to bedside. Nature 470:204–13 21307933 10.1038/nature09764

[23] Haga SB. 2010. Impact of limited population diversity of genome-wide association studies. Genet. Med. 12:81–84 20057316 10.1097/GIM.0b013e3181ca2bbf

[24] Halbert CH, Allen CG, Jefferson M, Magwood GS, Melvin C, 2020. Lessons learned from the Medical University of South Carolina transdisciplinary collaborative center (TCC) in precision medicine and minority men’s health. Am. J. Men’s Health 14:1557988320979236 33319609 10.1177/1557988320979236 PMC7745579

[25] Halbert CH, Brewster K, Collier A, Smith C, Kessler L, 2005. Recruiting African American women to participate in hereditary breast cancer research. J. Clin. Oncol. 23:7967–73 16258097 10.1200/JCO.2004.00.4952

[26] Halbert CH, Gandy OH Jr., Collier A, Shaker L. 2007. Beliefs about tobacco use in African Americans. Ethn. Dis. 17:92–98 17274216

[27] Halbert CH, Jefferson M, Allen CG, Babatunde OA, Drake R, 2021. Racial differences in patient portal activation and research enrollment among patients with prostate cancer. JCO Clin. Cancer Inform. 5:768–74 34328797 10.1200/CCI.20.00131 PMC8812608

[28] Halbert CH, Kessler L, Collier A, Weathers B, Stopfer J, 2012. Low rates of African American participation in genetic counseling and testing for BRCA1/2 mutations: racial disparities or just a difference? J. Genet. Couns. 21:676–83 22790832 10.1007/s10897-012-9485-y PMC3773724

[29] Halbert CH, Kessler L, Stopfer JE, Domchek S, Wileyto EP. 2006. Low rates of acceptance of *BRCA1* and *BRCA2* test results among African American women at increased risk for hereditary breast-ovarian cancer. Genet. Med. 8:576–82 16980814 10.1097/01.gim.0000237719.37908.54

[30] Halbert CH, Kessler L, Troxel AB, Stopfer JE, Domchek S. 2010. Effect of genetic counseling and testing for *BRCA1* and *BRCA2* mutations in African American women: a randomized trial. Public Health Genom. 13:440–48 10.1159/000293990 PMC3025895 20234119

[31] Halbert CH, Love D, Mayes T, Collier A, Weathers B, 2008. Retention of African American women in cancer genetics research. Am. J. Med. Genet. A 146A:166–73 18076114 10.1002/ajmg.a.32067

[32] Halbert CH, McDonald JA, Magwood G, Jefferson M. 2017. Beliefs about genetically targeted care in African Americans. J. Natl. Med. Assoc. 109:98–106 28599763 10.1016/j.jnma.2017.02.004 PMC5978775

[33] Halbert CH, McDonald JA, Vadaparampil S, Rice L, Jefferson M. 2016. Conducting precision medicine research with African Americans. PLOS ONE 11:e0154850 27441706 10.1371/journal.pone.0154850 PMC4956119

[34] Halbert CH, Stopfer JE, McDonald J, Weathers B, Collier A, 2011. Long-term reactions to genetic testing for *BRCA1* and *BRCA2* mutations: Does time heal women’s concerns? J. Clin. Oncol. 29:4302–6 21990416 10.1200/JCO.2010.33.1561 PMC3221529

[35] Halbert CH, Welch B, Lynch C, Magwood G, Rice L, 2016. Social determinants of family health history collection. J. Community Genet. 7:57–64 26280996 10.1007/s12687-015-0251-3 PMC4715811

[36] Hardiman G, Savage SJ, Hazard ES, da Silveira WA, Morgan R, 2021. A systems approach to interrogate gene expression patterns in African American men presenting with clinically localized prostate cancer. Cancers 13:5143 34680291 10.3390/cancers13205143 PMC8533960

[37] Hughes C, Fasaye GA, LaSalle VH, Finch C. 2003. Sociocultural influences on participation in genetic risk assessment and testing among African American women. Patient Educ. Couns. 51:107–14 14572939 10.1016/s0738-3991(02)00179-9

[38] Hughes C, Gomez-Caminero A, Benkendorf J, Kerner J, Isaacs C, 1997. Ethnic differences in knowledge and attitudes about BRCA1 testing in women at increased risk. Patient Educ. Couns. 32:51–62 9355572 10.1016/s0738-3991(97)00064-5

[39] Hughes C, Lerman C, Lustbader E. 1996. Ethnic differences in risk perception among women at increased risk for breast cancer. Breast Cancer Res. Treat. 40:25–35 8888150 10.1007/BF01806000

[40] Hughes C, Peterson SK, Ramirez A, Gallion KJ, McDonald PG, 2004. Minority recruitment in hereditary breast cancer research. Cancer Epidemiol. Biomark. Prev 13:1146–55 15247125

[41] Huo D, Senie RT, Daly M, Buys SS, Cummings S, 2009. Prediction of *BRCA* mutations using the BRCAPRO model in clinic-based African American, Hispanic, and other minority families in the United States. J. Clin. Oncol. 27:1184–90 19188678 10.1200/JCO.2008.17.5869 PMC2667822

[42] James RD, Yu JH, Henrikson NB, Bowen DJ, Fullerton SM. 2008. Strategies and stakeholders: minority recruitment in cancer genetics research. Community Genet. 11:241–49 18417972 10.1159/000116878

[43] Kaphingst KA, McBride CM, Wade C, Alford SH, Reid R, 2012. Patients’ understanding of and responses to multiplex genetic susceptibility test results. Genet. Med. 14:681–87 22481132 10.1038/gim.2012.22 PMC3417078

[44] Kessler L, Collier A, Halbert CH. 2007. Knowledge about genetics among African Americans. J. Genet. Couns. 16:191–200 17333408 10.1007/s10897-006-9054-3

[45] Khoury MJ, Feero WG, Valdez R. 2010. Family history and personal genomics as tools for improving health in an era of evidence-based medicine. Am. J. Prev. Med. 39:184–88 20621267 10.1016/j.amepre.2010.03.019

[46] Kinney AY, Steffen LE, Brumbach BH, Kohlmann W, Du R, 2016. Randomized noninferiority trial of telephone delivery of *BRCA1*/*2* genetic counseling compared with in-person counseling: 1-year follow-up. J. Clin. Oncol. 34:2914–24 27325848 10.1200/JCO.2015.65.9557 PMC5012661

[47] Krakow M, Rising CJ, Trivedi N, Yoon DC, Vanderpool RC. 2020. Prevalence and correlates of family cancer history knowledge and communication among US adults. Prev. Chronic Dis. 17:E146 33211995 10.5888/pcd17.200257 PMC7735478

[48] Lerman C, Hughes C, Benkendorf JL, Biesecker B, Kerner J, 1999. Racial differences in testing motivation and psychological distress following pretest education for *BRCA1* gene testing. Cancer Epidemiol. Biomark. Prev. 8:361–67 10207641

[49] Lerman C, Narod S, Schulman K, Hughes C, Gomez-Caminero A, 1996. *BRCA1* testing in families with hereditary breast-ovarian cancer: a prospective study of patient decision making and outcomes. JAMA 275:1885–92 8648868

[50] Lewis JP, Palmer ND, Hicks PJ, Sale MM, Langefeld CD, 2008. Association analysis in African Americans of European-derived type 2 diabetes single nucleotide polymorphisms from whole-genome association studies. Diabetes 57:2220–25 18443202 10.2337/db07-1319 PMC2494685

[51] Lynch JA, Berse B, Rabb M, Mosquin P, Chew R, 2018. Underutilization and disparities in access to *EGFR* testing among Medicare patients with lung cancer from 2010–2013. BMC Cancer 18:306 29554880 10.1186/s12885-018-4190-3 PMC5859516

[52] Mai PL, Vadaparampil ST, Breen N, McNeel TS, Wideroff L, 2014. Awareness of cancer susceptibility genetic testing: the 2000, 2005, and 2010 National Health Interview Surveys. Am. J. Prev. Med. 46:440–48 24745633 10.1016/j.amepre.2014.01.002 PMC4042677

[53] Manswell Butty JA, Richardson F, Mouton CP, Royal CD, Green RD, 2012. Evaluation findings from genetics and family health history community-based workshops for African Americans. J. Community Genet. 3:1–12 22109910 10.1007/s12687-011-0068-7 PMC3266965

[54] McBride CM, Wade CH, Kaphingst KA. 2010. Consumers’ views of direct-to-consumer genetic information. Annu. Rev. Genom. Hum. Genet. 11:427–46 10.1146/annurev-genom-082509-141604 20690815

[55] McCarthy AM, Bristol M, Domchek SM, Groeneveld PW, Kim Y, 2016. Health care segregation, physician recommendation, and racial disparities in *BRCA1*/*2* testing among women with breast cancer. J. Clin. Oncol. 34:2610–18 27161971 10.1200/JCO.2015.66.0019 PMC5012689

[56] McDonald JA, Vadaparampil S, Bowen D, Magwood G, Obeid JS, 2014. Intentions to donate to a biobank in a national sample of African Americans. Public Health Genom. 17:173–82 10.1159/000360472 PMC5019027 24942180

[57] McDonald JA, Weathers B, Barg FK, Troxel AB, Shea JA, 2012. Donation intentions for cancer genetics research among African Americans. Genet. Test Mol. Biomark. 16:252–58 10.1089/gtmb.2011.0119 PMC3326272 22224593

[58] Moorman PG, Skinner CS, Evans JP, Newman B, Sorenson JR, 2004. Racial differences in enrolment in a cancer genetics registry. Cancer Epidemiol. Biomark. Prev. 13:1349–54 15298957

[59] Nanda R, Schumm LP, Cummings S, Fackenthal JD, Sveen L, 2005. Genetic testing in an ethnically diverse cohort of high-risk women: a comparative analysis of *BRCA1* and *BRCA2* mutations in American families of European and African ancestry. JAMA 294:1925–33 16234499 10.1001/jama.294.15.1925

[60] Natl. Compr. Cancer. Netw. 2022. NCCN Guidelines: non-small cell lung cancer. Guidel. Version 1.2022, Natl. Compr. Cancer Netw, Plymouth Meeting, PA. Available at https://www.nccn.org/guidelines/guidelines-detail?category=1&id=1450

[61] O’Neill SM, Rubinstein WS, Wang C, Yoon PW, Acheson LS, 2009. Familial risk for common diseases in primary care: the Family Healthware Impact Trial. Am. J. Prev. Med. 36:506–14 19460658 10.1016/j.amepre.2009.03.002

[62] Orlando LA, Hauser ER, Christianson C, Powell KP, Buchanan AH, 2011. Protocol for implementation of family health history collection and decision support into primary care using a computerized family health history system. BMC Health Serv. Res. 11:264 21989281 10.1186/1472-6963-11-264 PMC3200182

[63] Payne PW Jr., Royal C. 2007. The role of genetic and sociopolitical definitions of race in clinical trials. J. Am. Acad. Orthop. Surg. 15:S100–4 17766781 10.5435/00124635-200700001-00021

[64] Penchansky R, Thomas JW. 1981. The concept of access: definition and relationship to consumer satisfaction. Med. Care 19:127–40 7206846 10.1097/00005650-198102000-00001

[65] Popejoy AB, Fullerton SM. 2016. Genomics is failing on diversity. Nature 538:161–64 27734877 10.1038/538161a PMC5089703

[66] Powell IJ, Carpten J, Dunston G, Kittles R, Bennett J, 2001. African-American heredity prostate cancer study: a model for genetic research. J. Natl. Med. Assoc. 93(Suppl.):25S–28S 11798061 PMC2719991

[67] Rebbeck TR, Sankar P. 2005. Ethnicity, ancestry, and race in molecular epidemiologic research. Cancer Epidemiol. Biomark. Prev. 14:2467–71 10.1158/1055-9965.EPI-05-0649 16284364

[68] Reid RJ, McBride CM, Alford SH, Price C, Baxevanis AD, 2012. Association between health-service use and multiplex genetic testing. Genet. Med. 14:852–59 22595941 10.1038/gim.2012.52 PMC3424345

[69] Royal C, Baffoe-Bonnie A, Kittles R, Powell I, Bennett J, 2000. Recruitment experience in the first phase of the African American Hereditary Prostate Cancer (AAHPC) study. Ann. Epidemiol 10:S68–77 11189095 10.1016/s1047-2797(00)00194-0

[70] Rubinstein WS, Acheson LS, O’Neill SM, Ruffin MT IV, Wang C, 2011. Clinical utility of family history for cancer screening and referral in primary care: a report from the Family Healthware Impact Trial. Genet. Med. 13:956–65 22075527 10.1097/GIM.0b013e3182241d88 PMC3425444

[71] Ruffin MT IV, Nease DE Jr., Sen A, Pace WD, Wang C, 2011. Effect of preventive messages tailored to family history on health behaviors: the Family Healthware Impact Trial. Ann. Fam. Med. 9:3–11 21242555 10.1370/afm.1197 PMC3022039

[72] Sankar P, Cho MK, Mountain J. 2007. Race and ethnicity in genetic research. Am. J. Med. Genet. 143:961–70 10.1002/ajmg.a.31575 PMC2271134 17345638

[73] Schwartz MD, Kaufman E, Peshkin BN, Isaacs C, Hughes C, 2003. Bilateral prophylactic oophorectomy and ovarian cancer screening following *BRCA1*/*BRCA2* mutation testing. J. Clin. Oncol. 21:4034–41 14581427 10.1200/JCO.2003.01.088

[74] Schwartz MD, Lerman C, Brogan B, Peshkin BN, Halbert CH, 2004. Impact of *BRCA1*/*BRCA2* counseling and testing on newly diagnosed breast cancer patients. J. Clin. Oncol. 22:1823–29 15067026 10.1200/JCO.2004.04.086

[75] Schwartz MD, Lerman C, Brogan B, Peshkin BN, Isaacs C, 2005. Utilization of BRCA1/BRCA2 mutation testing in newly diagnosed breast cancer patients. Cancer Epidemiol. Biomark. Prev. 14:1003–7 10.1158/1055-9965.EPI-03-0545 15824179

[76] Shabo Shvo A 2013. The patient-centric translational health record. Pharmacogenomics 14:349–52 23438879 10.2217/pgs.13.11

[77] Shields AE, Crown WH. 2012. Looking to the future: incorporating genomic information into disparities research to reduce measurement error and selection bias. Health Serv. Res. 47:1387–410 22515190 10.1111/j.1475-6773.2012.01413.x PMC3418832

[78] Spratt DE, Chan T, Waldron L, Speers C, Feng FY, 2016. Racial/ethnic disparities in genomic sequencing. JAMA Oncol. 2:1070–74 27366979 10.1001/jamaoncol.2016.1854 PMC5123755

[79] Theisen V, Duquette D, Kardia S, Wang C, Beene-Harris R, Bach J. 2005. Blood pressure Sunday: introducing genomics to the community through family history. Prev. Chronic Dis. 2:A23 PMC1327717 15888234

[80] Thompson HS, Valdimarsdottir HB, Duteau-Buck C, Guevarra J, Bovbjerg DH, 2002. Psychosocial predictors of *BRCA* counseling and testing decisions among urban African-American women. Cancer Epidemiol. Biomark. Prev. 11:1579–85 12496047

[81] Valdez R, Yoon PW, Qureshi N, Green RF, Khoury MJ. 2010. Family history in public health practice: a genomic tool for disease prevention and health promotion. Annu. Rev. Public Health 31:69–87 20070206 10.1146/annurev.publhealth.012809.103621

[82] Verma V, Haque W, Cushman TR, Lin C, Simone CB II, 2019. Racial and insurance-related disparities in delivery of immunotherapy-type compounds in the United States. J. Immunother. 42:55–64 30628924 10.1097/CJI.0000000000000253

[83] Wang C, Gallo RE, Fleisher L, Miller SM. 2011. Literacy assessment of family health history tools for public health prevention. Public Health Genom. 14:222–37 10.1159/000273689 PMC2891255 20090283

[84] Wash. Post, Kaiser Fam. Found., Harv. Univ. 2011. Race and recession survey. Rep., Wash. Post, Washington, DC; Kaiser Fam. Found., San Francisco, CA; Harv. Univ., Cambridge, MA. https://www.kff.org/wp-content/uploads/2013/01/8159-t.pdf

[85] Weathers B, Barg FK, Bowman M, Briggs V, Delmoor E, 2011. Using a mixed-methods approach to identify health concerns in an African American community. Am. J. Public Health 101:2087–92 21330592 10.2105/AJPH.2010.191775 PMC3107882

[86] Weathers B, Kessler L, Collier A, Stopfer JE, Domchek S, Halbert CH. 2009. Utilization of religious coping strategies among African American women at increased risk for hereditary breast and ovarian cancer. Fam. Community Health 32:218–27 19525703 10.1097/FCH.0b013e3181ab3b53 PMC4155033

[87] White House Pres. Barack Obama. 2016. Precision Medicine Initiative. White House: President Barack Obama. https://obamawhitehouse.archives.gov/precision-medicine

